# The One Material

**Published:** 1894-09

**Authors:** Max Greenbaum

**Affiliations:** Philadelphia


### THE ONE MATERIAL.

BY MAX GREENBAUM, D.D.S., PHILADELPHIA.

THE desire for an ideal filling-material is not new, as attempts towards such end have been made repeatedly. With each result the cry came forth afresh that at last the material was here with which all cavities should be filled, however disappointment followed. The work involved in these attempts is enormous, and it seems as though it should have been productive of a closer approach to the requirements. But mastery of difficulties may be impossible at times, and even though results point to disappointment, when we consider their relation to the whole requirements, still they may be very gratifying, and the mastery which seems impossible in its complete form is effected in part. This is the condition in dentistry, and an analysis of this condition is our concern.

Before complying with this, we must recognize the powerful relation it holds to the great dividing line among practitioners, both sides of which are held as being scientific. A consideration of this becomes necessary for the sake of completeness. Nor is this all. Not alone that our purpose here embraces a consideration of the different materials as they relate to an ideal conception, together with the great controversy involved in the different estimations in which they are held, but also the elimination of a grave error. If both sides of a question are advanced respectively as being scientific, there is an apparent contradiction. Both sides cannot be scientific, and, as the strongest safeguard a question can have is a claim to a scientific basis, the matter requires adjustment. Let us then borrow aid wherever we can, in order to advance the end.

The first effort is to ascertain what side of the controversy, as comprehended in the differences between the old and new school in dentistry, can be called scientific. But before we can do that, we must understand what is meant by the term science or scientific. By the term science we signify knowledge founded upon self-evident truths, or upon a basis that simulates so closely a self-evident truth that it stands as an accepted fact. Arithmetic is a system of classified knowledge founded upon evident truisms. Whatever the result may be from any of these truisms, it is accepted and admits of no deviation. That is science in its strictest sense. But we have a more liberal understanding of what the term science expresses that yet fulfils its meaning, as may be instanced by chemistry. The chemist deals with matter, but matter is no self-evident truth. In fact its ultimate expression cannot be comprehended; still the chemist, through experimentation, or by employing knowledge established and verified by means of experimentation, brings out new facts which also are classified as being scientific. This is the more liberal acceptation of the term,—knowledge gained through experimentation. It transpires sometimes that a fact belonging to a certain category of knowledge becomes subservient to the establishment of a fact, or facts, grouped under another division. Let us keep that in mind. Understanding, then, the meaning of the terms science and scientific, already a second question presents itself,—Which side of the great controversy is truly scientific? As it stands, the work of Dr. Palmer arranges itself on one side, and "defective manipulation" on the other. Now, both cannot be right, and therefore should not remain. An impartial examination, with whatever aid we can bring to it, should eliminate one or the other, and as the final result free our profession from such defects as the controversy implies.

Since teeth have been filled with gold its skilful manipulation has been considered *the* great attainment. The object has been the perfect introduction of difficult fillings,—the more difficult and nearer perfect the higher the standing of the dentist. This served as a powerful stimulus to the employment of foil; but after awhile a great many fillings failed, and as each failure presented itself the conclusion was reached that defective manipulation alone was the cause. Now, what does this explanation contain that can enable us to say of it, it is scientific? Is there anything appertaining to it that is founded upon a self-evident truth, or upon experimentation? Nothing. It is a mere surmise. We have no proof that such filling was introduced in a faulty manner, and therefore no right to assume so, providing we wish to keep within scientific bounds. But wishing to arrive at the truth, we admit the possibility of the filling having been introduced in a faulty manner, and in order to ascertain whether such was the case, we adopt a method.

We select an easy cavity, fill it perfectly, as we can do with great care and ability, and after completion feel thoroughly satisfied as to its perfection. We make a note of it; after three, four, or five years, as the case may be, we examine this filling and find it is not, by far, as perfect as when it was first introduced; we cannot say here defective manipulation is the cause of the imperfection. However, we do not feel satisfied with the result of one filling; we try several more, and, insisting upon the truth in the question, seek the co-operation of other practitioners. All conclude to introduce fillings, as well as can be done in easy places and with more than average skill, and observe the result. After several years, as the patients return, the first observation is confirmed,—the fillings return and they are not by far as well as when first introduced. To persist in the face of such evidence to explain these results by the cry of "defective manipulation" is absurd, to say the least. All this has been done, and still the "cry" persists. From beginning to end it is seen to be either an unfounded surmise, or else an answer framed in greatest error, both devoid of such procedures as are requisite to formulate a scientific explanation. Another argument may be adduced to strengthen the final one, why the cry of "defective manipulation" should be eradicated. It is historical that first answers usually prove erroneous. Were it not generally so accepted, numerous instances could be given how explanations in respect to gravest questions were compelled to adapt themselves to later discoveries, in some instances being entirely controverted. Here, too, then, there is a similarity that tends to relegate the cry of "defective manipulation" to an insufficient and erroneous state.

What merit does the second explanation contain? It is a result of investigations. Contrary to the first answer, which upon analysis was found to be mere guess work, or else an erroneous committal, the second is an impartial result of investigations directed to the purpose. However, it may be argued that even investigations, though scientific, if founded upon a false basis, produce false results, which is a fair criticism. What remains, then, is the demonstration that the basis is intact, which, together with scientific procedure, must bring about scientific results; and if scientific, they are correct, and in being correct supersede those that may antagonize their general or detailed expression. The best way to demonstrate a basis to be intact is to compare it with knowledge that stands accepted in the strictest sense of the word,—about which there is not the slightest antagonism,—or to apply it variously; and if this can be done consistently it certainly stands confirmed, and the least we can say is to pronounce it belonging to a very high order of knowledge, and one that is clothed in general verity. If it can be proved, then, that the basis contained in the second answer agrees with established knowledge, or can be applied variously with consistency, the least we can say of it is to pronounce it a very high order of knowledge, which is by itself far in advance of what we can say of the cry of "defective manipulation."

It is current among those whose business it is to know of such things that all particles of matter possess molecular motion. As our knowledge concerning matter is simply an approximation, for in its ultimate form it is beyond our understanding, we dare not construe this to be a definite, visible motion. Again, when we consider the fact, as held, that "matter is but an expression of force, under certain conditions in relation to our intelligence" (Spenser), which is the broadest conception we can frame, or the Newtonian conception as consisting of centres of attractive and repulsive forces, which is not as broad, together with the doctrines that "force is the very ultimate conception intelligence can frame," and that "matter and motion are concrete corellations of it" (Spenser), the assumption of molecular motion is a veritable deduction. Furthermore, if we admit into the argument the truism that "every particle of matter is at every moment going either from a point of integration to one of disintegration, or conversely, concomitant with which, in the first place, there is gain of motion, and in the second place loss of motion" (Spenser), it furnishes additional data for the conception of molecular motion. These are truisms expressing that unification of scientific knowledge which is the highest testimony to their legitimate nature. But another statement of a law must be observed,—*particles on coming in contact with each other produce such redistributions, concomitant with which certain motion must take place, or a certain expenditure of force.* Now, this is the truth we are seeking, and with it the work of Dr. Palmer harmonizes. Having set forth in the beginning what constitutes a scientific procedure, the value contained therein, together with the fact that if a result be applied variously, and serves with consistency in all applications, it is the highest evidence for its acceptance, and, furthermore, that all positive affairs are governed by these conditions, thereby securing unity of knowledge and correct interpretations, what now remains is the observation that the deduction of Palmer's work expressed in incompatibility of filling-material with tooth structures coincides with the general truth found; then all points converge to that centre of harmony which expresses the strongest evidence of their verity.

The redistribution of matter and consequent expenditure of force upon particles coming in contact with each other is identical to the disintegration of tooth-structure around fillings and consequent evolution of a galvanic current.

It must be understood that the current or force attending redistribution does not disintegrate tooth-structure, but aids in forming an increased amount of acids through secondary decomposition, and the increased amount of acids expedites failure.

This, then, is the fundamental agreement expressing that harmony which is the strongest evidence of its verity. To controvert the statement that disintegration is the inevitable result when fillings are introduced into cavities is equal to the attempt to antagonize the truism of redistribution of matter, and to antagonize that is to gainsay the accepted doctrine among foremost scientists.

Upon slight notice the foregoing may appear irrelevant to the title subject; however, an attempt to define respectively the positions of filling-materials must embrace a consideration of tooth-saving ability, and as great difference exists in opinion upon this point it became needful to effect some adjustment. The foregoing establishes the truth in the question, which is that gold, although lauded at one time as the material with which all cavities should be filled, to-day occupies a much lower position. The truisms with which we set out express unification of the highest scientific knowledge; if, then, they do not support the above statement, the statement is not true. However, just how much everything is in accord, it is only necessary to point out that the malleting of foil, which is imperative for its proper introduction, results in greater redistribution than without it. Every blow is accompanied by molecular rearrangement, and with every molecular rearrangement there is a corresponding expenditure of force. This is the simple expression. But there must be a compound expression. The molecular arrangement of the gold itself must undergo change through the force employed in inserting it, aside from the rearrangement of the structure of the tooth. This acts upon itself and reacts upon tooth-structure, and conversely. It is evident, then, that gold produces great molecular rearrangement, and consequently great expenditure of force, and as this is analagous to the disintegration of tooth-structure and failure of fillings, and depends in degree upon the structure of the tooth, what we stated as being the truth in the question without confirmation becomes the truth with it.

We may now pass on to the subject proper. There are other considerations that negative gold from the ideal stand-point,—it is not easy to introduce into cavities. This presents a two-fold argument; with the necessary care and skill attending it comes a wear upon the organization of the operator which cannot exist long without some break,—one of our ablest operators died at a comparatively early age from the excessive wear attending the use of gold. Then, too, the discomfort to which some patients are subjected are contrary considerations. Not infrequently complaints concerning the irritation upon application of the dam, or forceful separation of teeth, precludes work for the time being, or even a second attempt at the operation; especially is this so with highly nervous temperaments.

Conductivity and inability to be matched to the color of the tooth. Conductivity cannot be a quality of an ideal material. Such delicate sensibilities as displayed by the pulp render conductivity highly objectionable. Too many patients complain of the pain from thermal changes after the insertion of gold fillings, even though care may have been taken to employ pulp protectors, but where protection is rendered by substances that do not touch all parts of the cavity, the operator having deemed it expedient to apply the protection over the region of the pulp alone. Inability to be matched to the color of the tooth makes any material unideal. The ideal material must be one that can be finished with enamel lustre, and until a permanent preparation possess this quality, along with the others, we are unable to speak of an ideal material.

So far our considerations concerning gold have been of a negative nature. Let us see now what merit it has. Permanency under favorable conditions; this requires no extended comment; of all the attributes, permanency stands conspicuous. No matter in what respects a material is esteemed valuable, if it possess no permanency it stands, generally speaking, beneath those materials that have permanency; and gold under certain conditions is permanent. In hard-structured teeth, where the malleting of foil is not accompanied with such redistributions, and in consequence with no such expenditure of force as in soft-structured teeth, gold is a permanent filling. A good gold filling in such a tooth is certainly a permanent affair, lasting as long as the tooth remains in its socket. It is such teeth that have established the high position of gold as a filling-material. Non-shrinking and non-expanding are attributes of the ideal material and of gold. When we consider that much of the odium attached to amalgam arises from the crevicing due to its spheroiding tendency, although it can be remedied with the greatest ease, thereby securing a most valuable filling, we feel the importance of these attributes; and gold possesses them. By the foregoing considerations can be grouped resisting capability; the importance of that is self-evident; as so many cavities are situated in places exposed to attrition, we must look upon ability to resist attrition as an essential requisite of a material; although marked by qualities that justify the high position gold occupies, still the material is far beneath the ideal one. Its relative position among other materials usually employed may be noted more fully as the other materials are compared with the ideal attributes.

By the side of gold may be grouped tin-foil; with the exception of tooth-saving attribute, which with tin-foil is far greater than gold, and resisting capability, which is far less, its other qualities are so alike to those of gold that no separate examination is necessary.

The permanency disclosed by gold fillings under favorable circumstances contrasts so decidedly with the temporary service fulfilled by cement fillings that it seems in place to consider the cements next. The only preparation used as a filling-material is the zinc phosphate, and this, too, has its favorable and unfavorable aspects. It is comparatively easy to mix, easy to introduce into the cavity, adhesive to cavity-walls, has sufficient plasticity for continued manipulation, hardens with sufficient rapidity after finishing, offers resistance to attrition, is non-shrinking and non-expanding, is compatible with tooth-structure, non-conducting, and can be matched to the color of the tooth. In all these respects zinc phosphate is the ideal material. Easy to mix and easy to introduce into a cavity are attributes which have been considered in connection with gold. There we found the contrary to obtain, with its detriments; here we have them as attributes, with their advantages; adhesive to cavity-walls. Very frequently hypersensitive places require attention that cannot be shaped to retain any filling; in such places zinc phosphate, through its adhesive qualities, can be used. Then again, when we consider the great care with which cavities must be prepared to properly retain other fillings, and even in spite of accurate preparations fillings work themselves loose, we may further appreciate the advantage in the ability of the filling-material to adhere to the walls of the cavity. The plasticity of zinc phosphate, together with the degree of rapidity in hardening, are qualities that redound to its advantage. Although zinc phosphate sets quickly, after a time we learn to manipulate it so handily that the filling is introduced, properly trimmed and smoothed, and then pronounced set; and there is no material which we can introduce into a similar cavity and call it finished in the same length of time. The degree with which it hardens is considerable, giving it good body to resist attrition.

Non-shrinking and non-expanding, as stated in connection with gold, become the more important when we observe the criticisms hurled upon amalgams because some of them shrink. The ideal material necessarily does not shrink nor expand, and zinc phosphate insomuch has further claim to ideal distinction. Compatible with tooth-structure, this quality is the basis of all calculations. The conscientious exponent of the profession has for his motto tooth-salvation, and with no material can teeth be saved unless those are employed of which we can say, they are compatible with tooth-structure. The length at which we discussed the cause of failure in filling-operations may become now more obvious. It is the starting-point, and gives a material the valid claim to the distinction of being a filling-material while no other quality will do as much; non-conducting attribute and the ability to finish with enamel lustre are two stand-points from which zinc phosphate appears highly commendable. The irritation manifested through the conductivity of metal fillings at times results in serious complications, and occasionally necessitates the drilling out of recently introduced fillings and substitution of others with better pulp protectors; although zinc phosphate does not finish with enamel lustre, yet it can be matched to the shade of the tooth accurately; in fact, approaches the color of teeth better than any material in use with the exception of porcelain, and that we do not yet consider among our generally-employed materials for stopping teeth, so that we can say of zinc phosphate, it approaches the color of teeth better than any filling-material we employ. In all these respects zinc phosphate stands near to the ideal material. So many qualities appear in its favor, that from casual observation it may be wondered that there is any doubt as to its highest position among filling-materials, but we have to consider simply one deficiency, and it sinks to the lowest position, and that is its solubility in the fluids of the mouth; all the high respects in which zinc phosphate may be entertained are counter-balanced by its disintegration in the oral fluids, and ranks simply as a valuable adjunct. In spite of all that has been done to overcome this, practically very little has been accomplished. Zinc phosphate fillings dissolve in the oral fluids as much to-day as ever before. Should something be introduced to eliminate the deficiency to succumb to oral fluids, then indeed it will appear as though we had the ideal material. The disintegration of zinc phosphate fillings is not the only charge against them; there is another, although less important,—the liability to irritate the dental pulp. Many teeth have lost their vitality through the irritation of such fillings when the pulp-tissue was not properly protected; but with proper care this can be overcome entirely. And next within these considerations comes gutta-percha. In contrast to the former material here we have a substance that under favorable circumstances cannot be surpassed in permanency. In places where no other material seems able to maintain the integrity of the tooth, gutta-percha can be depended upon; and, as this is our essential point, gutta-percha holds correspondingly a very essential place in our list of filling-materials. As we found gold unworthy of the precedence accorded it, because it cannot save those teeth that require saving most, so is there ground for holding gutta-percha in very high estimation. Upon inquiry we ascertained that gold fillings resulted in early failure, because of the greater redistribution consequent upon its apposition with tooth-structure. So here we find gutta-percha, in its introduction into cavities, free from the force employed in the insertion of gold, which is sufficient to account for lesser redistributions and greater ability to maintain the integrity of the dentinal tissue.

Of the other attributes belonging to the ideal material, gutta-percha can lay claim to some: softens readily under heat for proper introduction; can be easily introduced into cavities; is non-irritating to the pulp; is non-conducting; can be matched to the shade of the tooth; is nearly insoluble in the fluids of the mouth. Some of these qualities require no further recommendation. The easy introduction of gutta-percha may be remarked upon. This does not signify introduction as the procedure is generally understood, with temporary stopping. Many gutta-percha fillings are introduced by heating the material over the spirit lamp and then pushing it into the cavity, and the name of a filling is bestowed upon such operation. When we say gutta-percha can be introduced easily, it is from the comparative stand-point; although inserted piece by piece, yet there are no thoughts about perfect adaptation and perfect condensation. It appears strange when we observe how one material may lack in a certain attribute,—as zinc phosphate, for instance, lacks in its inability to withstand the oral fluids, which condemns it completely, while otherwise it is almost ideal; and then another material possessing largely the ability to resist the oral fluids, on the other hand, lacks in other directions, which renders it quite deficient in comparison with the ideal requirements. So far we had the commendable attributes of the gutta-percha. Let us view the non-commendable ones. Gutta-percha is deficient in two respects,—non-resistance to attrition and leakage. The first is an irreparable fault; the second is of no detriment whatever, excepting the slight clouding that results to the tooth. The grave deficiency of gutta-percha presented in its failure to resist attrition necessarily restricts the use of it to such places as are without attrition. Few as the places are for the legitimate use of this material, yet when they arise we have the assurance that its employment will save the tooth as no other will. Then, again, cavities in soft-structured sensitive teeth, even though they implicate the coronal surface, may yet be filled with gutta-percha to advantage. The renewal is less worthy of consideration than the tooth-saving quality. And last of all we consider amalgam, because, of all the materials we have for filling teeth, amalgam is *the material*, possessing the greatest number of attributes from the ideal standpoint, and *the one material* which, through choice, we alone could select with which to maintain a practice. There is no material we have to-day which can be employed by itself in a practice and do one-half as well as amalgam, and no greater argument can be adduced for it.

This highest position assigned to amalgam must be adequately sustained, or else it is forced to a lower rank. The same line of argument that demonstrates gold cannot save soft teeth; and that gutta-percha can do so shows that certain amalgams can do the same. The molecular rearrangement of matter and incident motion when two particles are brought in apposition with each other, which upon analysis proved to be analogous with the disintegration of tooth-structure, and which under condition of gold and tooth-structure is greatest, because of the greater rearrangement of matter consequent upon the introduction of foil into the cavity, so under the condition of amalgam and tooth-structure this molecular rearrangement is considerably lessened, owing to the ease with which amalgam may be introduced.

This established the first important consideration,—tooth-saving ability. Then, too, it must be observed that if any force result from molecular rearrangement, it is invited upon the amalgam, thus saving the tooth, as darkened surfaces of fillings clearly show. The next important attribute which, owing to its absence, renders gutta-percha far from being the material, is resistance to attrition. Perhaps the importance of this attribute becomes more marked as we recognize its absence or presence. Could we speak of gutta-percha as being able to resist attrition, then indeed it would be the material for filling teeth in place of amalgam; but as it cannot withstand attrition, so it is forced to give precedence to amalgam. Amalgam, although introduced in the plastic form, rapidly becomes hard, and then we can say of it that it is eminently capable of offering resistance to attrition. This establishes its second important attribute. And the third is its insolubility in the fluids of the mouth. Here again we notice the importance of this quality as we recall that zinc phosphate is almost the ideal material, with the exception that it disintegrates in the oral fluids; and that, owing to this fact, it descends to a position where at best we can only speak of it as a valuable adjunct.

Tooth-conserving ability, resistance to attrition, and insolubility in the fluids of the mouth are the three essential qualifications of a material, the great tripod together enabling a material to maintain the front rank, while without any one, not enabling it to do so. Of the three materials, gold, gutta-percha, and zinc phosphate, each is wanting in one. Gold lacks in the first important attribute,—tooth-saving ability,—and the grounds upon which this rests are in accord with the highest scientific knowledge; while that upon which it is controverted appears as mere assumption, as was shown. Thus we have the choice in accepting between the highest scientific knowledge or an uncertainty as the guide in practice. Gutta-percha is deficient in offering resistance to attrition,—about this there is a unison of opinion,—while zinc phosphate dissolves in the oral fluids; this, too, is universally admitted. As we analyze the worth of amalgam, we must agree it is the material among filling-materials; because it is the only one that has claim to all three highest attributes. It requires no further argument to establish the front rank for amalgam, to the impartial and scientific mind. Besides these three attributes, amalgam bears other recommendations. It is easy to mix and easy to introduce into cavities. Even though any other material could be compared to amalgam from the stand-point of the three highest attributes, if it were not easy to mix and easy to introduce into cavities it would necessarily be compelled to give precedence to amalgam. When we speak of amalgam as being easily mixed, it signifies, nevertheless, the knowing how; different conditions require different treatments; in all cases accuracy and method underlie procedure. The ease of introduction bears more extended remarks; amalgam is easily introduced, providing knowledge is utilized. It is a matter of very little concern, after incorporating mercury with the alloy, to introduce this plastic result into the cavity, but to bring about best results is another matter. It necessitates accurate preparation of cavity, and accurate manipulation of material. Many early failures could be attributed to want of knowledge in preparation of cavity.

The ability to soften after setting and reuniting with a fresh mix is worthy of consideration. Very frequently complications render it expedient to remove a filling, and yet such removals are attended with severe pain, unless performed in an exceedingly gentle manner. Drilling out fillings under such circumstances, as would be the only course were gold the filling, is unnecessary where we have amalgam fillings. By a proper manipulation of soft amalgam, such fillings are easily removed and the pain incident to such removals necessarily lessened. Amalgam, being far from the ideal material, thus possesses qualities that are undesirable, and first is shrinkage or leakage. Here, too, we may add that moisture, external or internal, is associated with discoloration. As noted before, the molecular rearrangement of matter upon particles coming in contact with each other and consequent evolution of force, being analogous to the disintegration of tooth-structure and formation of a current, the disintegration is lessened when the force evolved exercises itself upon the filling. This is what we have when a darkened surface displays itself upon the filling; in so much, then, it is advantageous.

Conductivity is another charge against amalgam. Serious as the objections are to it, the same must be made against gold. Although the conductivity of silver is greater than that of gold, the combination with other metals diminishes it considerably; so that it is less than that of gold. Then, again, pulp-protectors are more readily adapted in operations where amalgam is employed than in those where the filling-material is gold. The conditions under which each is worked make this evident. No futher comment can be made against amalgam. Clearly it has been shown that among our filling-materials it is in the front rank. The work from beginning to end has for its sole aim the construction of a scientific basis for dentistry; and that basis begins with the acceptance of amalgam as the material of all our filling-materials. Appearances are sacrificed for tooth-salvation. That is the practice with the followers of the scientific in our profession. Gold enthusiasts accept the contrary. Their basis is æstheticism. But science works with a wonderful hand. Doctrine and practice are set aside when it proclaims the truth. It has begun its work in dentistry, and ere long we shall see its full exercise. We claim that until some new material possessing all the attributes of the ideal filling shall have been evolved, amalgam must take first rank.

